# Two *Acinetobacter baumannii* Isolates Obtained From a Fatal Necrotizing Fasciitis Infection Display Distinct Genomic and Phenotypic Characteristics in Comparison to Type Strains

**DOI:** 10.3389/fcimb.2021.635673

**Published:** 2021-04-12

**Authors:** Jennifer T. Grier, Brock A. Arivett, Maria S. Ramírez, Renee J. Chosed, Jessica A. Bigner, Emily J. Ohneck, Maeva L. Metz, Cecily R. Wood, Sergio Arce, Andrea Tartaro, Ryan F. Relich, Luis A. Actis, Steven E. Fiester

**Affiliations:** ^1^ Department of Biomedical Sciences, University of South Carolina School of Medicine Greenville, Greenville, SC, United States; ^2^ Department of Biology, Middle Tennessee State University, Murfreesboro, TN, United States; ^3^ Department of Biological Science, California State University Fullerton, Fullerton, CA, United States; ^4^ Department of Microbiology, Miami University, Oxford, OH, United States; ^5^ Cancer Institute, Prisma Health, Greenville, SC, United States; ^6^ Computer Science Department, Furman University, Greenville, SC, United States; ^7^ Department of Pathology and Laboratory Medicine, Indiana University School of Medicine, Indianapolis, IN, United States; ^8^ Department of Pathology, Prisma Health, Greenville, SC, United States

**Keywords:** *Acinetobacter*, necrotizing fasciitis, genomics, virulence phenotypes, XDR

## Abstract

*Acinetobacter baumannii* has been recognized as a critical pathogen that causes severe infections worldwide not only because of the emergence of extensively drug-resistant (XDR) derivatives, but also because of its ability to persist in medical environments and colonize compromised patients. While there are numerous reports describing the mechanisms by which this pathogen acquires resistance genes, little is known regarding *A. baumannii*’s virulence functions associated with rare manifestations of infection such as necrotizing fasciitis, making the determination and implementation of alternative therapeutic targets problematic. To address this knowledge gap, this report describes the analysis of the NFAb-1 and NFAb-2 XDR isolates, which were obtained at two time points during a fatal case of necrotizing fasciitis, at the genomic and functional levels. The comparative genomic analysis of these isolates with the ATCC 19606^T^ and ATCC 17978 strains showed that the NFAb-1 and NFAb-2 isolates are genetically different from each other as well as different from the ATCC 19606^T^ and ATCC 17978 clinical isolates. These genomic differences could be reflected in phenotypic differences observed in these NFAb isolates. Biofilm, cell viability and flow cytometry assays indicate that all tested strains caused significant decreases in A549 human alveolar epithelial cell viability with ATCC 17978, NFAb-1 and NFAb-2 producing significantly less biofilm and significantly more hemolysis and capacity for intracellular invasion than ATCC 19606^T^. NFAb-1 and NFAb-2 also demonstrated negligible surface motility but significant twitching motility compared to ATCC 19606^T^ and ATCC 17978, likely due to the presence of pili exceeding 2 µm in length, which are significantly longer and different from those previously described in the ATCC 19606^T^ and ATCC 17978 strains. Interestingly, infection with cells of the NFAb-1 isolate, which were obtained from a premortem blood sample, lead to significantly higher mortality rates than NFAb-2 bacteria, which were obtained from postmortem tissue samples, when tested using the *Galleria mellonella in vivo* infection model. These observations suggest potential changes in the virulence phenotype of the *A. baumannii* necrotizing fasciitis isolates over the course of infection by mechanisms and cell processes that remain to be identified.

## Introduction

The Gram-negative opportunistic human pathogen *Acinetobacter baumannii* is recognized by the Centers for Disease Control and Prevention and the World Health Organization as an urgent threat to public health and a critical priority for which the development of new antibiotics is crucially needed, respectively (Tacconelli et al., 2018; Moubareck and Halat, 2020). Infections with *A. baumannii* isolates resistant to carbapenems, antibiotics commonly used for the treatment of infections caused by this pathogen, resulted in 8,500 hospitalizations and 700 deaths in the United States alone during 2017 with approximately 281 million dollars in associated healthcare costs. This bacterial pathogen is most commonly associated with severe nosocomial infections including pneumonia, urinary tract infections, and sepsis (Wong et al., 2017). However, *A. baumannii* has also been associated with wound infections in military personnel deployed to the Middle East subsequently resulting in reports of *A. baumannii* infections in health care facilities that received wounded troops from Iraq (Scott et al., 2004; Davis et al., 2005; Scott et al., 2007). While the overall common clinical manifestations, as well as bacterial and host factors involved in the development of these infections have been described, the literature reporting the virulence properties and factors that participate in the pathogenesis of less common clinical manifestations including necrotizing fasciitis are lacking. Necrotizing fasciitis caused by *A. baumannii* is particularly rare but reports of these infections have increased over the last decade. In fact, one of the first confirmed cases in which *A. baumannii* was identified as the sole microbial cause of human necrotizing fasciitis was reported in 2009 (Charnot-Katsikas et al., 2009). Since then, at least ten more cases of human necrotizing fasciitis with *A. baumannii* as the sole etiological agent have been described in the literature (Corradino et al., 2010; Salvador et al., 2010; Sullivan et al., 2010; Wagner et al., 2011; Clemente et al., 2012; DeMuro et al., 2012; Hachimi et al., 2013; Oymacı et al., 2014; Sinha et al., 2014; Nehme et al., 2018; Rebai et al., 2018; Goret et al., 2019; Matthews et al., 2019).

Necrotizing fasciitis is a rare condition involving necrosis of the skin, subcutaneous fat, and superficial and deep fascia that is potentially lethal if untreated (Green et al., 1996; Angoules et al., 2007). Certain factors predispose individuals to *A. baumannii* necrotizing fasciitis infection including other skin or soft tissue infections, trauma and immune-compromising conditions (Hasham et al., 2005; Angoules et al., 2007; Davoudian and Flint, 2012). Necrotizing fasciitis is often difficult to diagnose, since these infections begin in deep subcutaneous tissue and fascia; thus, symptoms such as the presence of an erythematous area of cellulitis should be addressed immediately, because the time to intervention is directly correlated with mortality rate, which may be as high as 25% for this disease (Bilton et al., 1998; Hasham et al., 2005; Angoules et al., 2007; Davoudian and Flint, 2012).

Necrotizing fasciitis infections that are monomicrobial versus polymicrobial in nature facilitate the identification of virulence factors produced by the causative agent responsible for this clinical manifestation. In particular, while virulence factors responsible for *A. baumannii* pathogenicity have been reported in the literature, those associated with necrotizing fasciitis have not been elucidated and only a few case studies describing this etiology appear in the literature. Recent reviews (Harding et al., 2018; Morris et al., 2019; Moubareck and Halat, 2020) focus on bacterial factors responsible for the pathogenicity of *A. baumannii* and its capacity to persist in nosocomial environments and express resistance to different antimicrobial agents; therefore, description of these numerous factors will not be belabored here within. Of potential interest in the context of necrotizing fasciitis however are pilus-associated virulence functions including (1) the type I chaperone-usher pilus system (Csu pili) implicated in biofilm formation on abiotic surfaces (Tomaras et al., 2003), (2) the Pap pili system thought to be involved in biofilm formation and maintenance (Marti et al., 2011; Eijkelkamp et al., 2014), (3) the PrpA type I chaperone-usher pili system involved in surface motility, pellicle formation and virulence (Wood et al., 2018), and (4) the type IV PilA pili responsible for twitching motility in the closely related *A. nosocomialis* (Harding et al., 2013). Unfortunately, none of these factors or a specific virulence factor have been associated with the pathogenesis of necrotizing fasciitis. This knowledge gap warrants the study of *A. baumannii* isolates responsible for the pathogenesis of necrotizing fasciitis that could provide novel insights into the biology of this human pathogen.

In this report, we characterize two *A. baumannii* isolates, one collected from a male patient’s bloodstream (NFAb-1) and the other (NFAb-2) obtained from the same patient’s postmortem tissue, originally reported as the sole causative agent of lethal necrotizing fasciitis by Charnot-Katsikas et al. (Charnot-Katsikas et al., 2009). The patient in this case had multiple underlying comorbidities including systemic lupus erythematosus, mesenteric vasculitis and thrombotic thrombocytopenic purpura with a prolonged course of corticosteroids. The extent of the necrotizing fasciitis was severe resulting in death within 36 h of the patient reporting initial pain in his flank and thigh. This infection was not only unusual because *A. baumannii* was the sole etiological agent associated with the presentation of necrotizing fasciitis, but also because of the rapid clinical course. It was proposed that these strains may represent the emergence of *A. baumannii* strain(s) with potentially enhanced virulence factors (Charnot-Katsikas et al., 2009), which had not previously been identified or characterized. To this end, the analysis of the NFAb-1 and NFAb-2 genome sequences, which we recently reported (Arivett et al., 2020), found both NFAb isolates to be genetically different from each other as well as from the clinical isolates ATCC 19606^T^, originally identified as the causative agent of an urinary tract infection (Schaub and Hauber, 1948), and ATCC 17978, which was isolated from a case of fatal meningitis in a 4-month old infant. The NFAb-1 and NFAb-2 isolates were also phenotypically compared to strains ATCC 19606^T^ and ATCC 17978. This comparative analysis showed that both NFAb strains display pronounced twitching motility and significant biofilm formation that may be related to the presence of pili longer than 2 µm. Both NFAb isolates displayed the capacity for intracellular invasion and were found to affect the viability of erythrocytes and respiratory epithelial cells, with one strain possessing a greater effect on host cell viability that correlates with increased virulence when tested using the *Galleria mellonella* infection model. In summary, our data indicate that although the NFAb-1 and NFAb-2 isolates share common genetic and phenotypic traits with two clinical ATCC strains isolated years ago from patients suffering different clinical symptoms, they have distinct properties even among themselves. These different properties could be related to the capacity of the NFAb-1 and NFAb-2 isolates to cause the severe and lethal soft tissue infection originally associated with these two *A. baumannii* isolates (Charnot-Katsikas et al., 2009).

## Material and Methods

### Identification, Antimicrobial Susceptibility Testing, and Growth Conditions of Bacterial Strains

All bacterial strains used in this work, which are listed in **Table 1**, were routinely maintained as Luria-Bertani (LB) broth/glycerol stocks. LB, Mueller-Hinton (MH) and cation-adjusted Mueller-Hinton broth (CAMHB) were used to propagate bacterial strains as specified. Chelex 100-treated Trypticase soy broth dialysate (TSBD) containing 10% horse erythrocytes was specifically used for hemolysis assays as described before (Fiester et al., 2016). Motility medium broth (MMB; 10 g tryptone and 5 g NaCl per liter of water) and motility medium agarose plates (MMA) (MMB with the addition of 0.3% agarose) were used to study surface and twitching motility. Unless otherwise indicated, bacterial cultures were incubated overnight (16 h) at 37°C with liquid cultures shaken at 200 rpm. Bacterial growth curves were determined from LB, MMB or MH broth cultures incubated at 37°C in a Tecan Infinite M1000 Pro microplate reader/incubator with intermittent double orbital shaking at 168 rpm (Tecan Group Ltd.). All growth assays were repeated in triplicate using fresh biological samples each time with two technical replicates for each repeat.

**Table 1:** Bacterial strains and plasmids used in this work.

*A. baumannii* strains	Relevant characteristic(s)	Source/reference
ATCC 19606^T^	Wildtype clinical isolate	ATCC
19606 #144	19606 *csuE*::EZTN <R6K*γori*/KAN-2> derivative, Km^R^, biofilm deficient	(Tomaras et al., 2003)
ATCC 17978	Wildtype clinical isolate	ATCC
NFAb-1	Blood isolate from case 1, patient X	(Charnot-Katsikas et al., 2009)
NFAb-2	Postmortem tissue-isolate from case 1, patient X	(Charnot-Katsikas et al., 2009)

Matrix-assisted laser desorption/ionization time-of-flight mass spectrometry (MALDI-TOF MS) was used to confirm the identity of bacteria isolated as the sole etiological agents of the case of necrotizing fasciitis described above using a Bruker microflex analyzer and MALDI Biotyper v3.1 software (Bruker Daltonic) as described before (Arivett et al., 2015). Antimicrobial and antibiotic susceptibility testing was performed using the Epsilometer test (Etest) to determine minimal inhibitory concentrations (MIC). The Etest (AB bioMérieux) was performed and interpreted according to the manufacturer’s instructions. Briefly, bacterial suspensions equivalent to a 0.5 McFarland standard were used to inoculate MH agar plates (Remel) prior to strip placement. Plates were incubated for 22 h in ambient air at 35°C prior to MIC interpretation. MICs were determined by recording the drug concentration on the strip at which bacterial growth intersected. Isolates’ susceptibility to doxycycline was performed by the Kirby-Bauer method according to standard procedures. Automated antimicrobial susceptibility test (AST) determinations were made using the VITEK 2 XL (bioMérieux) according to the manufacturer’s instructions. Interpretive criteria were based on CLSI breakpoints, with the exception of the gentamicin MIC for ATCC 19606^T^, where the European Committee on Antimicrobial Susceptibility Testing (EUCAST) breakpoints were used.

### Comparative Genomic Analyses

Bioinformatic analyses were performed using *A. baumannii* ATCC 19606^T^, ATCC 17978, NFAb-1 and NFAb-2 whole genomes retrieved from NCBI GenBank. Genome sequences were assembled using MaSuRCa (Zimin et al., 2013) and annotated with PROKKA (Seemann, 2014) to improve comparisons between genomes. Genomes annotated through the same pipeline were used in parallel analyses using the ROARY and BPGA pangenome analysis software packages (Page et al., 2015; Chaudhari et al., 2016). BlastKOALA (https://www.kegg.jp/blastkoala/) was used to determine KEGG annotations of proteins present in NFAb-1 and NFAb-2 (Kanehisa et al., 2016).

### Surface and Twitching Motility Assays

MMA plates were stab inoculated with bacteria lifted from overnight LB agar cultures using sterile inoculation needles. After incubation for 24 h at 37°C, motility plates were photographed to assess surface motility (Mussi et al., 2010). Previous to recording twitching motility, the agarose layer was removed from the plates, the bacteria attached to the plates were stained with 0.1% crystal violet (wt/vol) in water for 20 min, excess crystal violet was gently rinsed away with water, and plates were allowed to air dry (Harding et al., 2013). All surface and twitching motility experiments were repeated at least twice in triplicate using fresh biological samples each time. The areas to which bacteria moved on the surface of motility plates and twitched were ascertained using ImageJ.

### Biofilm Formation Assays

Biofilm formation on polystyrene tubes was assessed with modifications as previously described (Tomaras et al., 2003). Briefly, bacteria were statically cultured in MMB and stained with 1% crystal violet to assess biofilm or left unstained to assess growth. Crystal violet-stained cells attached to plastic tubes were thoroughly rinsed with distilled water, crystal violet was solubilized with ethanol-acetone as we described previously (Tomaras et al., 2003). The amount of biofilm formed was colorimetrically assessed and normalized to the total cell content of each sample using OD_580/600_ ratios. The ATCC 19606^T^ #144 isogenic mutant (**Table 1**) served as a negative control due to its inability to produce biofilms on polystyrene tubes as a result of impaired production of type I CsuABABCDE pili (Tomaras et al., 2003). Biofilm assays were performed in triplicate at least twice using fresh biological samples each time. Scanning electron micrographs were obtained from 5 ml-cultures incubated statically in MMB for 24 h in 50-ml conical tubes with semi-submerged polystyrene coverslips at 37°C. Coverslips were processed for scanning electron microscopy, as previously described (Tomaras et al., 2003). Biofilm ultrastructures were visualized using a Zeiss Supra Gemini 35VP field emission scanning electron microscope. Images above, at and below the air-liquid interface were captured at 5,000 x magnification at an accelerating voltage of 5 keV.

The capacity of *A. baumannii* strains to form biofilms was also tested using the macrocolony biofilm assay, which provides some information on the nature of the extracellular matrix including poly-N-acetylglucosamine (PNAG), amyloid curly and cellulose (Serra and Hengge, 2017; Wermser and Lopez, 2018). Briefly, assays were performed by inoculating LB agar plates supplemented with 40 µg/ml Congo red, 20 µg/ml Coomassie brilliant blue and 2 µg/ml collagen I with 5 µl of culture from each *A. baumannii* tested strain. Plates were incubated statically for 24 h at 37°C. The results were observed and recorded using a USB 2.0 Digital Microscope.

### Detection of PilA

The production of PilA, which is a type IV pilin produced by *A. nosocomialis* (Harding et al., 2013) was determined by western blotting using anti-PilA polyclonal rabbit antisera generated as described (Harding et al., 2015). Briefly, cells of the ATCC 17978, NFAb-1 and NFAb-2 strains were collected from the plastic surface of motility plates after the agarose layer of MMA plates was removed, while ATCC 19606^T^ cells, which do not display twitching motility, were collected from the surface of MMA plates. Cells were resuspended in sterile PBS and OD_600_ values were determined to normalize cell numbers prior to lysis. Cell pellets were then lysed in 100 µl of 5X SDS sample buffer (250 mM Tris-HCl, pH6.8; 10% SDS; 30% glycerol; 5% beta-mercaptoethanol and 0.02% bromophenol blue) and boiled for 5 minutes. Equal volumes of cell lysates were loaded onto 4-12% NuPAGE Bis-Tris gels (Invitrogen), transferred to Immobilon-PSQ PVDF membranes (Millipore). Membranes were incubated at 4°C with a 1:1,000 dilution of anti-PilA serum for 16 h. Immunocomplexes were detected by chemiluminescence using an 1:5,000 dilution of anti-rabbit IgG HRP-linked secondary antibody (GE Healthcare) and ECL Prime Western Blotting Detection Reagent (GE Healthcare).

### Transmission Electron Microscopy

MMA plates were stab inoculated and incubated overnight at 37°C. An approximately 2 cm x 2 cm square of MMA was cut and removed from the inoculation site to expose the bottom of the Petri dish. Then, 5 μl of ddH2O was added to the exposed bottom of the Petri dish and a nitrocellulose substrate, carbon-coated, 300 mesh copper grid was placed face down on the area covered by the water for approximately 30 seconds. Grids were removed and 2 μl of 1.5% ammonium molybdate was immediately added for 30 seconds. Excess stain was removed, and the cells were allowed to dry at room temperature. Imaging was performed on a JEOL JEM-1200 EX II transmission electron microscope at an accelerating voltage of 120 keV.

### Host Cell Viability Assays

Strains were incubated in TSBD containing 10% horse erythrocytes (Cleveland Scientific, Ltd.) for 20 h at 37°C with shaking at 200 rpm to assess the hemolytic activity of each strain (Fiester et al., 2016; Fiester et al., 2019). Briefly, each culture was diluted 1:1000 in erythrocyte wash buffer (20 mM KH_2_PO_4_, 60 mM Na_2_HPO_4_, 120 mM NaCl, pH 8.0) (Stoebner and Payne, 1988), and intact erythrocytes were quantified using forward and side scatter channels on an Attune NxT flow cytometer (ThermoFisher). To establish an analysis gate that included only intact erythrocytes and excluded cellular debris or bacterial cells, fresh erythrocytes were diluted in erythrocyte wash buffer and compared to control samples of erythrocytes incubated at room temperature for 15 minutes in High-Yield Lysis Buffer (Invitrogen) or an erythrocyte-free bacterial culture in TSBD, both of which were diluted in erythrocyte wash buffer prior to sample collection. The number of remaining erythrocytes following incubation with *A. baumannii* strains was reported as erythrocytes per milliliter.

The effect of ATCC 19606^T^, ATCC 17978, NFAb-1 and NFAb-2 on A549 human alveolar epithelial cell viability was quantified using the CellTiter-Glo assay kit (Promega) following manufacturer’s instructions as previously described (Fiester et al., 2016; Fiester et al., 2019). Briefly, Dulbecco’s modified Eagle’s medium supplemented with 10% heat-inactivated fetal bovine serum and 1% penicillin-streptomycin was used to propagate A549 cells in the presence of 5% CO_2_ at 37°C. A549 cells were passaged three times before seeding cells in Hank‘s balanced salts solution without glucose into a white, opaque, 96-well plate in the absence of antibiotics with 1 x 10^5^ A549 cells. Experimental wells were inoculated with 1 x 10^6^ bacteria, while control wells were left uninfected. Following incubation in 5% CO_2_ for 20 h at 37°C and a wash with DMEM, the CellTiter-Glo assay was used to determine A549 cell viability, which is reported as relative luminescence units (RLUs). CellTiter assays were repeated three times in triplicate using fresh biological samples each time.

### Epithelial Cell Invasion Assays

The ability of *A. baumannii* to invade and survive within A549 cells was assessed using protection assays as described previously (Giannouli et al., 2013) wherein the virulence-related traits of epidemic *A. baumannii* strains belonging to the international clonal lineages I-III and to the emerging genotypes ST25 and ST78 were assessed. Briefly 1.5 x10^5^ cells per well, in a 24-well plate, were grown in DMEM containing 10% fetal bovine serum without antibiotics overnight. Cells were washed twice with PBS then infected at a multiplicity of infection (MOI) of 100 in DMEM alone. Infections were synchronized by centrifugation for 5 min at 59 x *g* followed by incubation at 37°C with 5% CO_2_. After 2 h, cells were washed twice with PBS to remove non-adherent bacteria. Cultured cells were then treated with 50 µg/ml of colistin sulfate (Sigma) in DMEM with 10% FBS for 30 minutes, which resulted in killing of all extracellular bacteria for all strains tested. Due to the XDR phenotype of the NFAb isolates, colistin was used *in lieu* of the more commonly used gentamicin as described before because colistin is not transported into host cells (Sato et al., 2019). Afterwards, A549 cells were harvested with trypsin, washed with PBS, and lysed with 100 µl of sterile distilled water. Dilutions of A549 lysis supernatants were inoculated on LB agar plates and incubated overnight at 37°C before determination of colony counts. Three independent replicates were performed.

### *G. mellonella* Virulence Assays

*A. baumannii* cells were collected by centrifugation and resuspended in PBS. Appropriate bacterial inocula were estimated spectrophotometrically at OD_600_ and confirmed by plate counting using LB agar plates. To assess virulence, *G. mellonella* survival assays were performed by injecting 10 randomly selected healthy final-instar *G. mellonella* larvae (*n*=30) with 10^5^ CFUs/larva (± 0.5 log) of each bacterial strain on three different days as previously described (Gaddy et al., 2012). Controls included non-injected larvae or larvae injected with 5 μl of sterile PBS. If more than two deaths in a control group were observed, the trial was discontinued and repeated. After injection, the larvae were incubated at 37°C in a humidified chamber with no light. Death was assessed at 24-h intervals over 5 days with removal of dead larvae at the times of inspection.

### Statistical Analyses

Confidence in species-level identification using MALDI-TOF MS was considered high if score values were greater than 2.000. Growth characteristics over 12 h of incubation in LB, MH or MMB were analyzed using the Growthcurver open-source R package (Sprouffske and Wagner, 2016), which fits growth curve data to the “standard form of the logistic equation.” One-way analysis of variance (ANOVA) followed by Tukey Honest Significant Differences (Tukey HSD) pairwise comparison of means post-hoc tests conducted in R were used to compare intrinsic growth rate (r) and doubling time (t_DT_) returned from the growth rate analysis (Thanissery et al., 2017). Significant differences in twitching motility, biofilm formation and intracellular invasion were determined using one-way ANOVA and Tukey-Kramer multiple comparisons post-hoc tests provided as part of the GraphPad InStat software package (GraphPad Software, Inc.). Significance of surface motility data was determined using the Student’s *t*-test comparing the surface motility of ATCC 17978 cells, which was previously tested (Mussi et al., 2010), to the motility of ATCC 19606^T^, NFAb-1 or NFAb-2 cells. The Student’s *t*-test was also utilized to determine the significance of *A. baumannii* strains’ impact on red blood cell lysis and A549 cell viability (GraphPad Software, Inc.). For *G. mellonella* virulence assays, survival curves were plotted using the Kaplan-Meier method, and significance was determined using the log-rank test of survival curves (SAS Institute Inc., Cary, NC) (Kaplan and Meier, 1958). Statistical significance was set *a priori* at *P* ≤ 0.05 for all experimentation.

## Results

### Necrotizing Fasciitis Isolates Display XDR with Significant Differences in Growth as Compared to Type Strains

MALDI-TOF MS confirmed the identity of both bacterial strains isolated from a male with a lethal case of necrotizing fasciitis as *A. baumannii.* The first isolate obtained from blood was therefore designated NFAb-1 (Necrotizing Fasciitis *Acinetobacter baumannii* isolate 1), and the second isolate collected from postmortem tissue was designated NFAb-2 (Necrotizing Fasciitis *Acinetobacter baumannii* isolate 2). The MALDI-TOF MS score values were 2.528 and 2.461 for NFAb-1 and NFAb-2, respectively. These scores indicate a high level of confidence in the species identifications as proposed by Patel (2015) in his review regarding the use of this technology for the diagnosis of infectious diseases (Patel, 2015).

Antimicrobial susceptibility testing performed using the Etest and the VITEK^®^ 2 XL system showed resistance of both NFAb-1 and NFAb-2 to all tested therapeutics except for colistin to which both were susceptible, and tobramycin to which both were intermediate (**Table 2**). Doxycycline susceptibility testing using the Kirby-Bauer method demonstrated that both NFAb-1 and NFAb-2 were susceptible to this antibiotic as well (**Table 2**). These resistance phenotypes indicate that both NFAb-1 and NFAb-2 are XDR isolates.

**Table 2:** Antimicrobial susceptibilities of *A. baumannii* isolates.

Method	Antimicrobial	19606		17978		NFAb-1		NFAb-2	
		MIC^a^	CI^b^	MIC^a^	CI^b^	MIC^a^	CI^b^	MIC^a^	CI^b^
E-test	Ceftazidime	12	*	6	S	≥256	R	≥256	R
	Cefepime	24	*	3	S	64	R	≥256	R
	Ciprofloxacin	0.75	S	0.25	S	≥32	R	≥32	R
	Colistin	0.125	S	0.38	S	0.25	S	0.25	S
	Gentamicin	12	R	1.5	S	≥256	R	≥256	R
	Levofloxacin	0.5	S	0.19	S	≥32	R	≥32	R
	Meropenem	1.5	S	0.75	S	≥32	R	≥32	R
	Tigecycline	2	*	0.38	*	1.5	*	2	*
	Tobramycin	3	S	0.5	S	96	R	64	R
	Trimethoprim-sulfamethoxazole	≥640	R	≥640	R	≥640	R	≥32	R
VITEK-2	Ampicillin	≥32	R	≥32	R	≥32	R	≥32	R
	Ampicillin-sulbactam	≤2	S	≤2	S	≥32	R	≥32	R
	Cefazolin	≥64	R	≥64	R	≥64	R	≥64	R
	Cefepime	16	I	2	S	≥64	R	≥64	R
	Cefoxitin	≥64	R	≥64	R	≥64	R	≥64	R
	Ceftazidime	8	S	4	S	≥64	R	≥64	R
	Ciprofloxacin	0.5	S	≤0.25	S	≥4	R	≥64	R
	Ceftriaxone	16	I	16	I	≥64	R	≥4	R
	Gentamicin	4	S	≤1	S	≥16	R	≥16	R
	Levofloxacin	0.25	S	≤0.12	S	≥8	R	≥8	R
	Meropenem	1	S	≤0.25	S	≥16	R	≥16	R
	Nitrofurantoin	256	R	≥512	R	256	R	256	R
	Piperacillin-tazobactam	≤4	S	≤4	S	≥128	R	≥128	R
	Tobramycin	≤1	S	≤1	S	8	I	8	I
	Trimethoprim-sulfamethoxazole	160	R	160	R	≥320	R	≥320	R
Kirby-Bauer		ZOI^c^	CI^b^	ZOI^c^	Ci^b^	ZOI^c^	CI^b^	ZOI^c^	CI^b^
	Doxycycline	27	S	26	S	27	S	28	S

a Minimum inhibitory concentration (MIC) values are presented in micrograms per milliliter.

b The categorical interpretation (CI) of susceptibility are noted as follows: R, resistant; S, susceptible; I, intermediate; *, no interpretive criteria.

c Zone of inhibition (ZOI) diameters are reported in millimeters.

**Figure 1:**
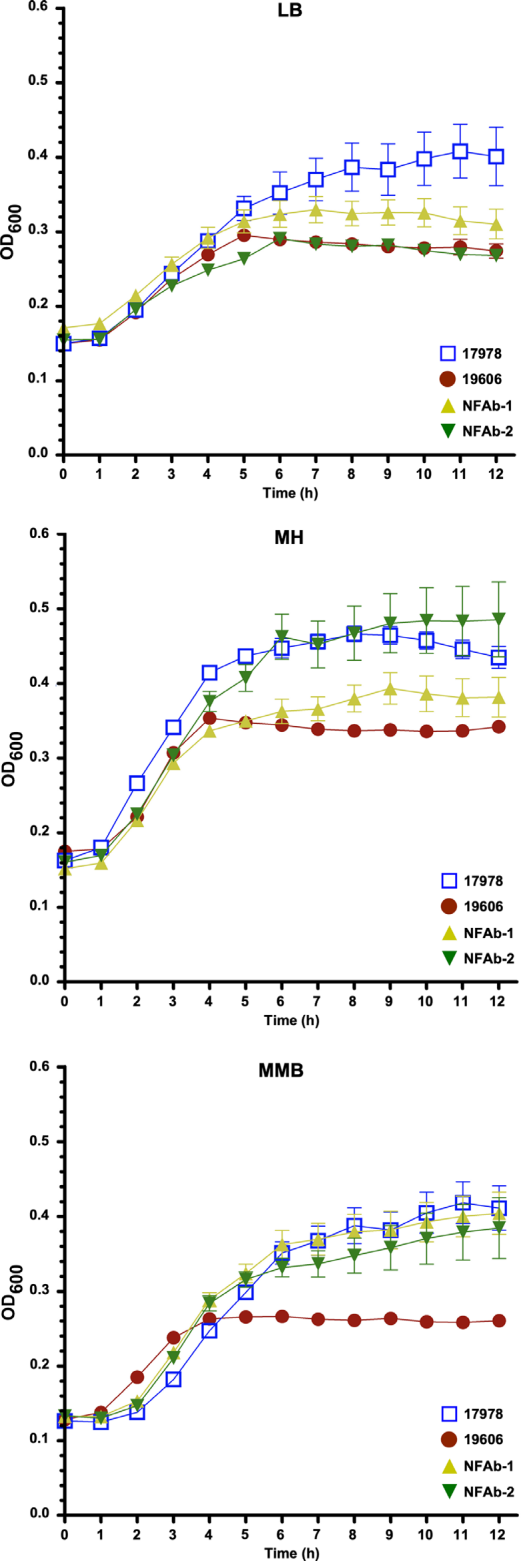
Growth of *A. baumannii* strains in different media. Growth curves of the ATCC 19606^T^, ATCC 17978, NFAb-1 and NFAb-2 strains cultured in LB, Mueller-Hinton (MH) or motility medium broth (MMB) at 37°C with shaking using three independent biological samples for each strain. The error bars represent the standard error of each data set.

Growth differences and similarities were observed when the ATCC 19606^T^, ATCC 17978, NFAb-1 and NFAb-2 strains were cultured at 37°C in LB, MH or MMB (**Figure 1**, **Supplemental Table S1**). In LB, there were no significant differences in growth rate or doubling time between NFAb-1 and NFAb-2. The growth rate of ATCC 17978 was however significantly lower than the growth rate of ATCC 19606^T^ (*P* < 0.001). The doubling time of ATCC 17978 was significantly higher than the doubling time of ATCC 19606^T^ (*P* < 0.001), NFAb-1 (*P* < 0.01) and NFAb-2 (*P* < 0.05). In MH, the growth rate of ATCC 19606^T^ was significantly greater than the growth rate of ATCC 17978 (*P* < 0.05), and the doubling time of ATCC 19606^T^ was significantly lower than the doubling time for NFAb-2 (*P* < 0.01). In MMB, the growth rate of ATCC 19606^T^ is significantly greater than the growth rate and ATCC 17978 (*P* < 0.01), NFAb-1 (*P* < 0.01) and NFAb-2 (*P* < 0.05), and the doubling time of ATCC 19606^T^ is significantly lower than that of ATCC 17978 (*P* < 0.05), NFAb-1 (*P* < 0.05) and NFAb-2 (*P* < 0.05). No other significant differences were observed. Taken together, these observations indicate that the NFAb-1 and NFAb-2 strains do not have striking growth differences when compared to the ATCC type strains 17978 and 19606^T^, with the latter showing the lowest growth in all three media under the experimental conditions used in this study.

### Necrotizing Fasciitis Isolates Display Genomic Diversity

**Figure 2:**
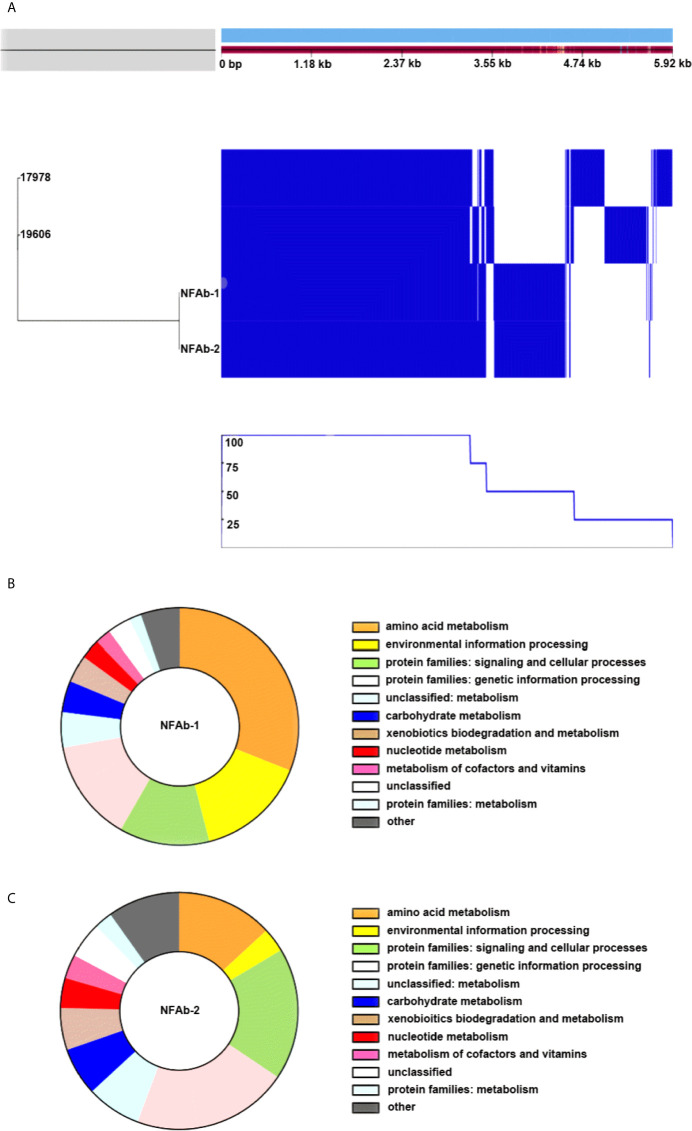
Comparative analysis of *A*. *baumannii* genomes*. A. baumannii* ATCC 19606^T^, ATCC 17978, NFAb-1 and NFAb-2 genomes were compared using ROARY and BlastKOALA. The relatedness of each isolate is seen at the left of **(A)** and the presence or absence of genes for each strain is seen in blue on the right with a corresponding line graph of the percent of strains harboring the above gene. The KEGG taxonomy analysis of NFAb-1 **(B)** and NFAb-2 **(C)** shows the predicted proteins that are unique to these isolates. The taxonomic groups are presented in the color-coded key to the right of each panel. **(A)** allows an estimation of the core genome shared between strains. The accessory genes of the necrotizing fasciitis isolates are further presented in **(B, C)** NFAb-1 has a greater percentage of proteins related to amino acid metabolism and environmental information processing than NFAb-2.

Pangenome analysis of *A. baumannii* ATCC 19606^T^, ATCC 17978, NFAb-1, and NFAb-2 demonstrated that 55.1% of genes analyzed, 2,916 out of 5,293 total genes, were core genes which were present in all four strains, while the remaining 44.9% of analyzed genes, 2,377 genes, were shell genes present in at least one strain (**Figure 2A**, **Supplemental Tables S2**–**S5**). Comparative analysis of gene presence by strain indicated that strains ATCC 19606^T^ and ATCC 17978 are more closely related to each other than to either NFAb-1 or NFAb-2 isolates (**Figure 2A**). Multiple gene clusters appear to be strain specific to the necrotizing fasciitis strains, implying that the NFAb-1 and NFAb-2 isolates differ significantly from the ATCC 19606^T^ and ATCC 17978 type strains, with additional strain-specific gene clusters present in ATCC 19606^T^ and ATCC 17978.

Further investigation of gene occurrence found greater than 1,000 genes that were only present in one of the analyzed *A. baumannii* strains, while 900 genes were present in only two of the strains (**Figure 2A**, **Supplemental Tables S2**–**S5**). NFAb-1 and NFAb-2 specifically share 846 genes not present in the ATCC 19606^T^ or ATCC 17978 genomes. Of those genes, 74% were annotated as coding for hypothetical proteins. Of the remaining 217 genes, 64% have annotations that are similar to genes in ATCC 19606^T^ and 57% have annotations similar to ATCC 17978 genes. KEGG Orthology (Kanehisa et al., 2016) and Links Annotation of protein-coding genes of NFAb-1 and NFAb-2 not present in ATCC 19606^T^ or ATCC 17978 genomes are shown in **Figures 2B, C**, respectively. The following taxonomy groups were identical between NFAb-1 and NFAb-2 protein families: genetic information processing, unclassified metabolism, carbohydrate metabolism, xenobiotic biodegradation and metabolism, nucleotide metabolism, metabolism of cofactors and vitamins, unclassified and protein families: metabolism (**Figure 2A**, **Supplemental Tables S2**–**S5**). There were variations between the necrotizing fasciitis isolates in the amino acid metabolism group, which included 58 and 42 protein-coding genes for NFAb-1 and NFAb-2, respectively, and the environmental information processing group wherein the NFAb-1 strain contained 28 protein-coding genes versus 4 genes in NFAb-2. Interestingly, the NFAb-2 genome contains 3 protein-coding genes compared to 1 in NFAb-1 with functions related to bacterial virulence. These two orthologies are k07347 and k0734, which are related to the type I pilus components FimA and FimD. Genes coding three modification methylases, BspRI, DpnIIA and RsrI, are present in both necrotizing fasciitis strains, as are genes coding for the mRNA interferases RelE and YafQ, potentially driving a unique expression profile of these isolates.

### Necrotizing Fasciitis Isolates Exhibit Differences in Motility and Biofilm Formation Compared to ATCC Strains

**Figure 3:**
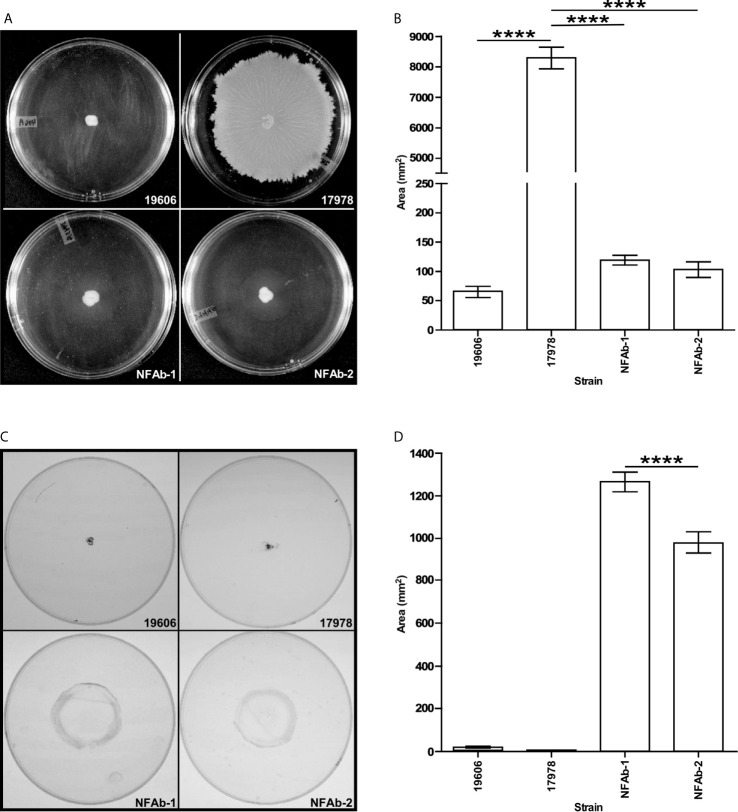
Surface and twitching motility of *A. baumannii* strains. MMA plates were stab inoculated with cells from the ATCC 19606^T^, ATCC 17978, NFAb-1 or NFAb-2 strains and incubated overnight at 37°C. After recording surface motility **(A, B)**, the agarose layers were removed, the plates were gently washed with distilled water and then stained with crystal violet to determine twitching motility **(C, D)**. Images in **(A, C)** display representative results while data presented in **(B, D)** were collected using three independent biological replicates with each of them tested in triplicate. ATCC 19606^T^, NFAb-1 and NFAb-2 cells displayed growth but not surface motility. Horizontal bars identify statistically different values (****P ≤ 0.0001) and error bars represent the standard error of each data set.

Inoculation and subsequent analysis of *A. baumannii* isolates on MMA plates demonstrated that ATCC 17978 cells moved significantly more (*P* < 0.0001) on the surface as compared to NFAb-1, NFAb-2 and ATCC 19606^T^ cells, all of which had relatively negligible surface motility (**Figures 3A, B**). Assessment of twitching motility *via* inoculation through MMA onto the surface of polystyrene Petri dishes showed that NFAb-1 and NFAb-2 cells were significantly more motile (*P* < 0.0001) when compared to either ATCC 19606^T^ or ATCC 17978 cells (**Figures 3C, D**). Interestingly, NFAb-1 displayed significantly more twitching motility than NFAb-2 (*P* < 0.0001).

**Figure 4:**
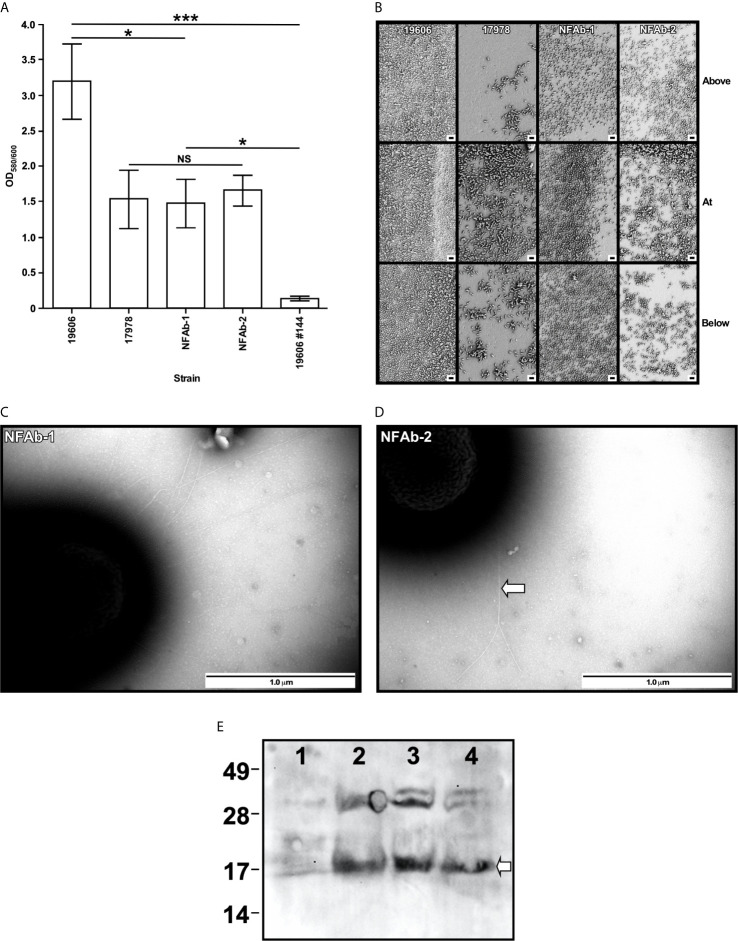
Biofilm formation, pili detection and PilA production by *A*. *baumannii* strains. **(A)** The formation of biofilms on polystyrene tubes was detected after cells were incubated statically in MMB overnight at 37C. OD_600_ was used to determine the total cell mass of each tested sample. OD_580_ was used to determine the amount of biofilm formed on plastic tubes after staining with crystal violet and solubilization with acetone-ethanol. Triplicates of three independent biological samples were used to collect and analyze experimental data. Horizontal bars identify statistically different values (*P* ≤ 0.05, *; *P* ≤ 0.001, ***, NS, not significant) and error bars represent the standard error of each data set. **(B)** SEM of biofilms formed on the surface of plastic coverslips semi-submerged in MMB and statically incubated overnight at 37°C. Samples were examined above, at and below the liquid-air interface. Images are representative of at least three micrographs collected at 5,000X magnification for each biological sample. The black bars at the bottom right side of each panel represent a 2 µm size. **(C, D)** TEM of cells lifted form the surface of the Petri dishes after the agarose layer was removed from MMA plates. The horizontal white arrow in panel D identifies entangled pili forming bundles. Images are representative of at least three micrographs collected at a 60,000X magnification for each biological sample. **(E)** Detection of PilA in ATCC 19606^T^ (lane 1), ATCC 17978 (lane 2), NFAb-1 (lane 3), and NFAb-2 (lane 4) total cell lysates. Numbers on the left indicate the position of molecular weight markers represented in kDa. The white arrow identifies the approximately 17-kDa PilA protein band.

Biofilm assays showed that the ATCC 17978, NFAb-1 and NFAb-2 strains produce significantly less (*P* < 0.05) biofilm on polystyrene than ATCC 19606^T^ when cultured statically in MMB at 37°C (**Figure 4A**). In contrast, there were no significant differences between the amounts of biofilm formed by ATCC 17978, NFAb-1 and NFAb-2. Macrocolony biofilm assays showed that although no significant differences were noted among all four tested strains, the edges of the NFAb-1 and NFAb-2 colonies, which were comparable, were slightly different to those displayed by the ATCC strains (**Supplemental Figure S1**). It was also noted that although they were able to bind Congo red and display the formation of exopolymers, none of them showed signs of structures such as wrinkled or rugose as it was described for other unrelated bacteria (Serra and Hengge, 2017; Wermser and Lopez, 2018).

The capacity of the tested isolates to form biofilms was further examined using SEM as described previously (Tomaras et al., 2003). This approach confirmed that ATCC 19606^T^ cells attached to the plastic surface at a much higher density and produced more cell aggregates at all three locations on the coverslips when compared to the structure and cell density of the biofilms produced by cells of the ATCC 17978, NFAb-1 and NFAb-2 strains (**Figure 4B**). The latter three strains produced their most dense and complex structures primarily at the air-liquid interface (**Figure 4B**).

Based on our observations that pili production is critical for biofilm formation and motility (Tomaras et al., 2003; Wood et al., 2018), the capacity of the NFAb-1 and NFAb-2 isolates to produce pili was examined by TEM. This approach showed that NFAb-1 and NFAb-2 indeed produce thin pili that are not only different in length and thickness from pili described for ATCC 19606^T^ (Tomaras et al., 2003) and ATCC 17978 (Tucker et al., 2014), but also remarkably long with some of them exceeding 2 µm in length. It should be noted however, that Tomaras et al. (2003) reported that ATCC 19606^T^ produced pili that were shorter and fairly distributed on the surface of bacteria collected from Tris-M9 agar plates, while Tucker et al. (2014) described the presence of shorter pili on the surface of ATCC 17978 cells collected from LB agar plates. It is apparent however that NFAb-1 cells produced more pili when compared with NFAb-2 cells, which tend to be associated with forming bundles (**Figures 4C, D**).

Since the most profound difference in NFAb motility and biofilm phenotypes was in regard to twitching motility, the production of the PilA protein, a type IV pilin originally described for *A. nosocomialis* and associated with *Acinetobacter* twitching motility (Harding et al., 2013; Tucker et al., 2014), was tested by immunoblotting of total cell lysate proteins. The western blot shown in **Figure 4E** demonstrates that the ATCC 17978, NFAb-1 and NFAb-2 strains produce the approximately 17-kDa protein that reacted with the anti-PilA antiserum that was described before during the identification of an oligosaccharyltransferase involved in the *O*-glycosylation of the *A. nosocomialis* M2 PliA protein (Harding et al., 2015). This protein band was not detectable in the ATCC 19606^T^ cell lysate (**Figure 4E**). Taken together, these observations indicate that the twitching motility phenotype of the necrotizing fasciitis strains could be related to the production of a type IV pilin such as PilA, although the production of this protein and the presence of *pil* orthologs found in the NFAb-1 and NFAb-2 strains as well as the ATCC 17978 isolate (**Supplemental Table S6**) are not the sole conditions for the expression of twitching motility. In contrast, the failure of ATCC 19606^T^ cells to display twitching motility directly correlates with lack of production of PilA and the absence of a gene coding for such a protein in the genome of this strain (**Supplemental Table S6**).

### *A. baumannii* Necrotizing Fasciitis Strains Have a Negative Effect on Host Cell Viability

**Figure 5:**
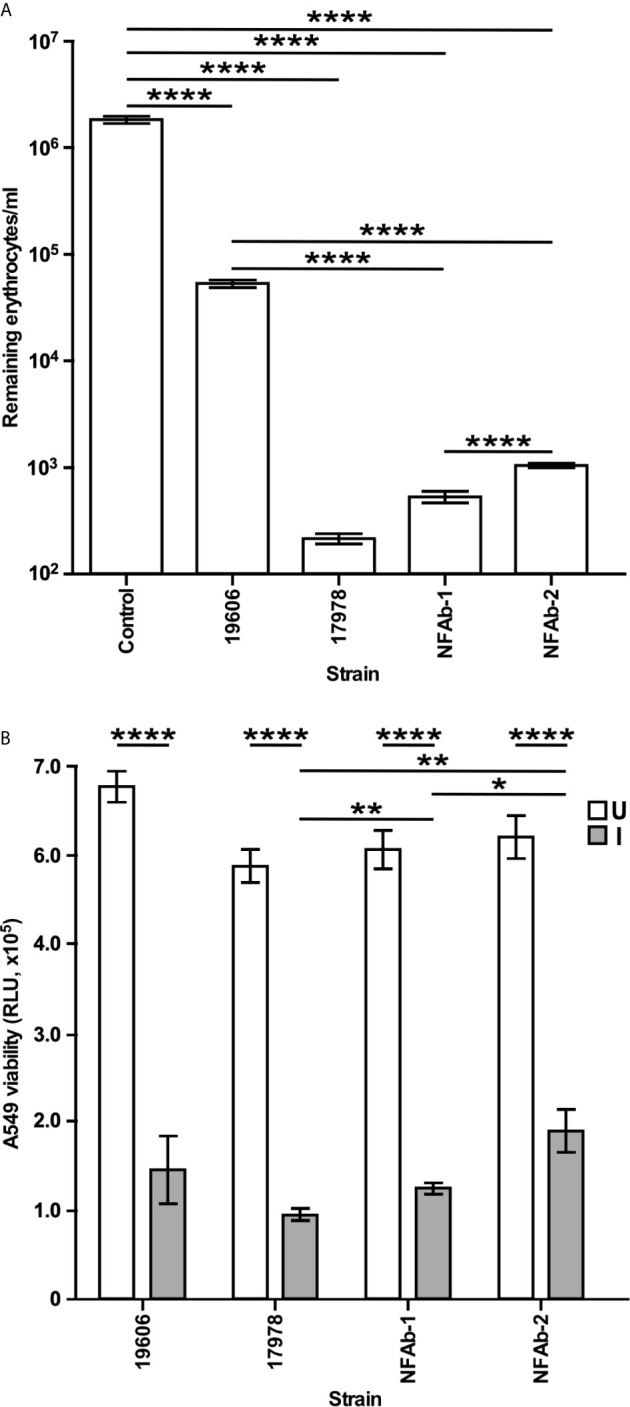
*A*. *baumannii* effect on host cell viability. **(A)** Hemolytic activity was tested by determining the number of horse erythrocytes remaining after incubation with cells of each of the *A. baumannii* strains at 37°C with shaking. **(B)** The effects of *A. baumannii* strains on A549 cell viability uninfected (U) or infected (I) with bacteria for 20 h at 37 °C with 5% CO2 was quantified using the CellTiter-Glo luminescent cell viability assay. The results, which are expressed as relative luminescence units (RLU), represent triplicates of three independent biological samples. Horizontal bars identify statistically different values (*P* ≤ 0.05, *; *P* ≤ 0.01, **; *P* ≤ 0.0001,****) and error bars represent the standard error of each data set.

Quantification of intact erythrocytes following incubation in metal-depleted media with *A. baumannii* isolates demonstrated significant hemolytic activity in all of the bacterial strains tested compared to a bacteria-free control (*P* < 0.0001, **Figure 5A**) with NFAb-1 and NFAb-2 causing over a 3-log reduction in intact cell numbers. Both necrotizing fasciitis strains were significantly more hemolytic than ATCC 19606^T^ (*P* < 0.0001) with NFAb-1 displaying a significantly higher activity than NFAb-2 (*P* < 0.0001); however, of the tested strains, ATCC 17978 was the most hemolytic with a nearly 4-log decrease in the concentration of intact erythrocytes when compared to bacteria-free erythrocyte cultures. Differences in growth between tested strains in TSBD at 20 h is noncontributory to the observed differences in hemolytic activity, the growth of all bacteria strains were within less than a log of one another (data not shown).

Since the NFAb strains are associated with infection of the epithelium and underlying tissue, they were tested for their effect on respiratory epithelial cell viability. Following infection of A549 human alveolar epithelial cells with *A. baumannii* bacteria over 20 h, A549 viability significantly decreased for all strains tested (*P* < 0.0001, **Figure 5B**). The necrotizing fasciitis strains were specifically found to diminish the viability of A549 cells with NFAb-1 leading to an approximately 75% decrease in A549 cell viability, while NFAb-2 reduced cell viability by roughly 64% (**Figure 5B**), indicating that both strains retained the ability to infect respiratory tissues, with NFAb-1 demonstrating significantly more virulence than NFAb-2 (*P* < 0.05). Notably, ATCC 17978 had significantly more of an effect on A549 cell viability than either NFAb-1 (*P* < 0.01) or NFAb-2 (*P* < 0.01).

### Intracellular Survival of Necrotizing Fasciitis *A. baumannii* Isolates in Respiratory Epithelial Cells

**Figure 6:**
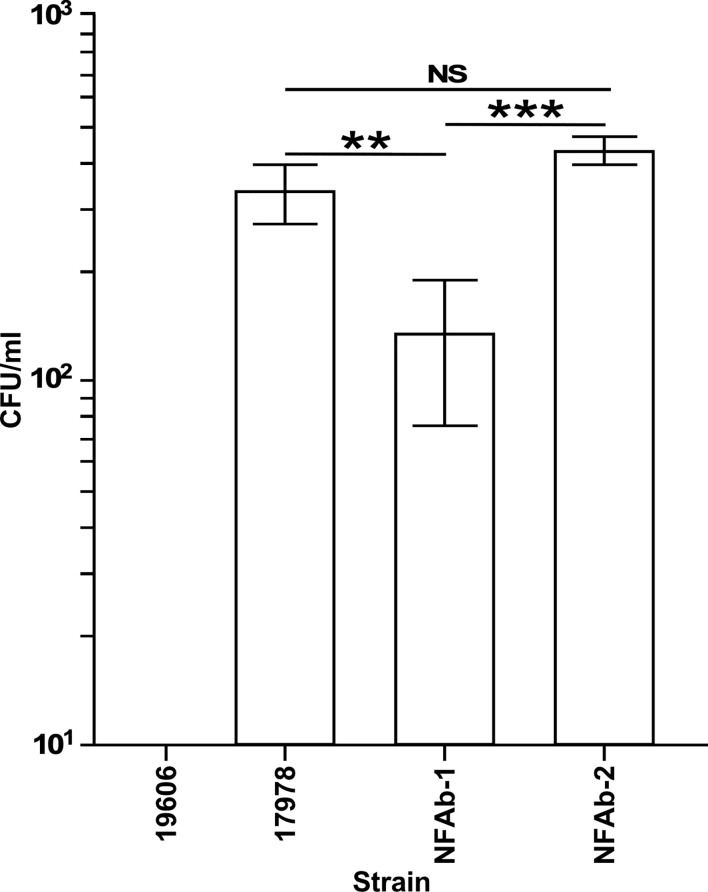
Intracellular invasion of *A. baumannii* strains into A549 respiratory epithelial cells. Surviving intracellular bacteria were collected after 2 hours of culture with A549 cells followed by a 30-min incubation in the presence of 50 μg/ml colistin. The number of bacteria surviving within A549 cells are reported as colony forming units (CFUs) obtained from triplicate independent biological samples. Horizontal bars identify statistically different values (*P* ≤ 0.01, **; *P* ≤ 0.001, ***; NS, not significant) and error bars represent the standard error of each data set.

The ability of *A. baumannii* strains to invade the respiratory epithelial cell line A549 was investigated by treating A549 cells with colistin after 2 h of infection at a MOI of 100 to determine intracellular bacterial counts. Both NFAb-1 and NFAb-2 demonstrated the ability to invade A549 cells with a greater number of NFAb-2 bacteria entering the intracellular compartment compared to NFAb-1 (*P* < 0.001), which was significantly less invasive than ATCC 17978 (*P* < 0.01) (**Figure 6**). Only ATCC 19606^T^ bacteria did not quantifiably invade the epithelial cells under the tested conditions.

### Virulence in *G. mellonella*

**Figure 7:**
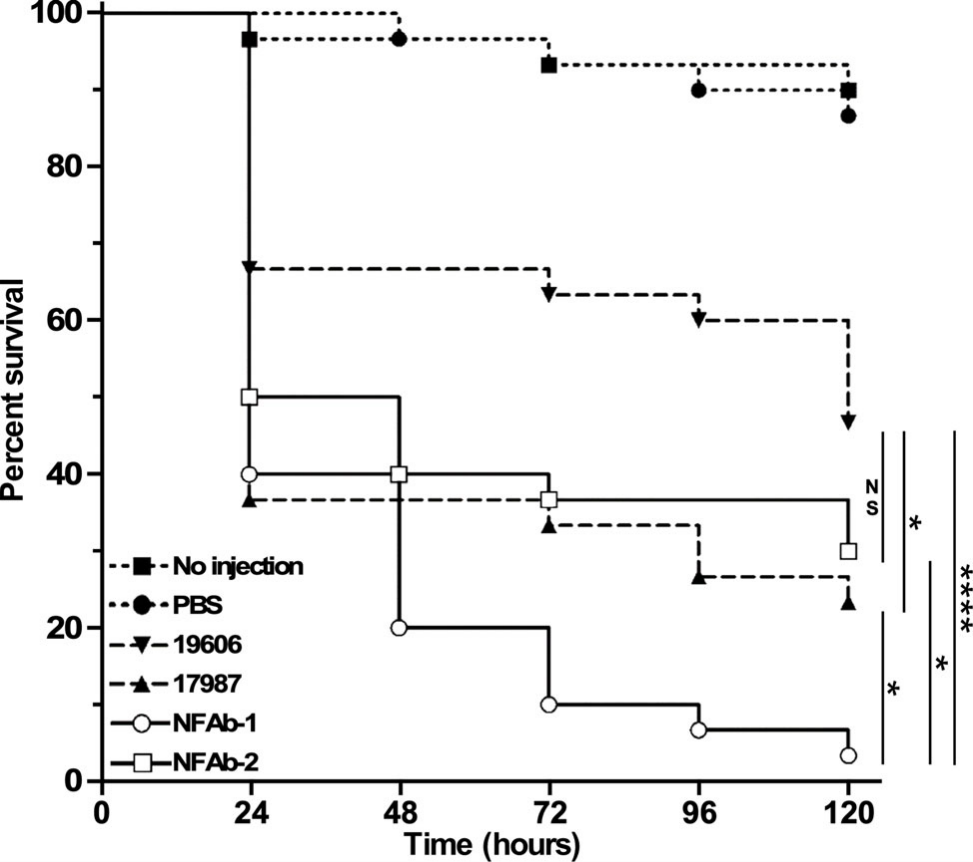
Virulence of different *A. baumannii* strains. *G. mellonella* larvae (*n=*30 per experimental condition) were injected with 10^5^ bacterial cells of each of the tested strains. Non-injected larvae and larvae injected with sterile PBS were used as negative controls. Larvae were incubated at 37°C and assessed for death at 24-h intervals over 120 hours (5 days). The control data (no injection and PBS injection) are significantly different from larvae injected with 19606 bacteria (*P* = 0.006) or 17978, NFAb-1 or NFAb-2 bacteria (*P* ≤ 0.0001). Vertical bars identify statistically different values (*P* ≤ 0.05, *; *P* ≤ 0.0001, ****; NS, not significant).

The virulence of the four *A. baumannii* strains used in this study was tested using *G. mellonella* as an invertebrate host that is capable of mounting a response similar to that described in vertebrate animals (Kavanagh and Reeves, 2004). Such a concept was confirmed during our analysis of *A. baumannii* ATCC 19606^T^ isogenic iron deficient mutants using the *G. mellonella* and mouse sepsis models; both of them proved that iron acquisition is indeed a critical virulence function for this pathogen (Gaddy et al., 2012). *G. mellonella* infections showed that the type strain ATCC 19606^T^ killed more than 50% of the infected larvae 120 h after they were infected (**Figure 7**), a response that not only is significantly different from that obtained with animals not injected or injected with sterile PBS, but also similar to that reported previously (Gaddy et al., 2012). The strains ATCC 17978 and NFAb-2 showed similar killing rates, however only ATCC 17978 exhibited significantly higher mortality (*P* = 0.0299) than that displayed by ATCC 19606^T^. Interestingly, the NFAb-1 isolate displayed the highest virulence when compared with all other strains with a killing rate higher than 90% five days after infection which was significantly greater than ATCC 19606^T^ (*P* < 0.0001). Taken together, these results indicate that the virulence properties between the necrotizing fasciitis isolates are as variable as those displayed by the ATCC strains, which are considered old isolates that are more susceptible to antibiotics and less virulent when compared with more contemporaneous clinical isolates.

## Discussion

The majority of *A. baumannii* cases have historically caused infections such as pneumonia, urinary tract infections and sepsis (Wong et al., 2017); therefore, the preponderance of *A. baumannii* research has focused upon strains representative of these more common types of infections. In comparison to antibiotic resistance properties of *A. baumannii*, the virulence phenotypes and factors expressed by this pathogen, particularly among different clinical isolates, are scantly elucidated. Additionally, of the few cases of necrotizing fasciitis caused by *A. baumannii* reported, none of them investigate the underlying virulence phenotypes and/or mechanisms utilized by this pathogen in this type of infection. Taken together this has resulted in a substantial gap in the understanding of the virulence mechanisms employed by *A. baumannii*, in general, and the virulence mechanisms utilized by this pathogen during rare emerging types of infections, in particular necrotizing fasciitis. These observations prompted us to initiate the virulence and genetic analyses of the NFAb-1 and NFAb-2 strains, which were isolated from a patient with a fatal case of necrotizing fasciitis. These strains were compared with the ATCC type strains 19606^T^ and 17978, both of which have been extensively used to study the pathobiology of *A. baumannii*. The NFAb strains proved to be XDR isolates susceptible only to colistin and doxycycline and intermediately resistant to tobramycin (**Table 2**). Of note, doxycycline is not commonly used to treat Gram-negative bacilli including *A. baumannii*, and using doxycycline monotherapy for the treatment of *A. baumannii* has proven ineffective therefore effectively leaving only one therapeutic option, colistin, for the treatment of infections caused by these XDR isolates (Rodriguez-Hernandez et al., 2000). 

Given the rapid clinical course of the infection described in the patient from which the NFAb isolates were recovered, as in many other cases of necrotizing fasciitis, bacterial growth rate could be an important consideration in understanding the virulence of these particular isolates. Furthermore, it has been previously suggested that antimicrobial resistance may decrease overall biological fitness (Beceiro et al., 2013). Our data, which show that the XDR strains NFAb-1 and NFAb-2 displayed growth rates that were not strikingly different from those of the ATCC 19606^T^ and 17978 strains (**Figure 1**), indicate that neither the progression of the infection nor the fitness of the NFAb XDR strains are apparently related to their growth phenotypes when compared to the ATCC strains under the experimental conditions used in this study. Notably, the ATCC 19606^T^ strain showed the lowest cell density when cultured for 12 h in all three liquid media, particularly MMB which is nutrient limited when compared to LB and MH. The growth differences that were observed could be explained by the results of our comparative analysis of the genomes of the NFAb isolates and the ATCC strains that suggest variable expression of genes involved in metabolic control (**Figures 2B, C**). These observations are in line with reported global genotypic and phenotypic diversity among *A. baumannii* isolates (Sahl et al., 2015), however studies of a variety of clinical *A. baumannii* sequence types from an Italian hospital found no significant difference in growth rates compared to ATCC 17978 (Ambrosi et al., 2017).

As the name implies, *Acinetobacter* was once considered to be a non-motile pathogen; however, many isolates do display motility. Bacterial motility has been closely linked with increased virulence and a pathogen’s ability to cause disease with *A. baumannii* being no exception. A hypermotile derivative of ATCC 17978 showed increased adherence to human cells and was significantly more lethal in a *Caenorhabditis elegans* model of infection (Eijkelkamp et al., 2013), whereas a mutant strain with impaired motility displayed only limited lethality when tested with this model (Perez-Varela et al., 2017). Each of these situations was revealed by changes in surface motility; however, some *A. baumannii* isolates, such as NFAb-1 and NFAb-2, are also capable of twitching motility (**Figure 3**), though the connections between twitching motility and virulence is not yet clear. Nevertheless, it is tempting to speculate that twitching motility is one of the bacterial factors that could contribute to the rapid and extensive progression of the tissue damage observed in this particular patient. Interestingly, it has been previously reported that *A. baumannii* blood isolates demonstrate increased twitching motility compared to respiratory isolates (Vijayakumar et al., 2016), which parallels the finding that greater twitching motility was demonstrated by NFAb-1, isolated from blood, than NFAb-2, isolated from postmortem tissue. Due to the numerous virulence factors produced by *A. baumannii*, many of which remain to be characterize in general and none of which have been associated with the pathogenesis of necrotizing fasciitis in particular, it is not possible at this stage to specifically attribute the difference in twitching motility between NFAb-1 and NFAb-2 to differences in pathogenicity. It is likely that factors in addition to twitching motility play roles in the pathogenicity of these strains. It should be noted however that increased twitching motility paralleled the increased virulence of NFAb-1 when incubated with red blood cells or used to infect A549 epithelial cells (**Figure 5**) or *G. mellonella* larvae (**Figure 7**) compared to NFAb-2 bacteria, an experimental observation suggesting a critical role for twitching motility in pathogenicity. While differences between *A. baumannii* isolates using *in vitro* assays are statistically significant, these differences may appear to have modest biological significance. We submit however that the biological significance between these isolates are more clearly delineated and obvious using the *in vivo G. mellonella* experimental infection model. Taking into account all these observations, it is possible to speculate that the distinct genotype and phenotype of the NFAb isolates, when compared to the ATCC strains, relate to the type of infection these isolates cause in the human host. This possibility remains to be tested using an adequate animal model such as the murine model developed to study the pathogenesis of streptococcal necrotizing fasciitis (Keller et al., 2018).

Type IV pili assemble into narrow fibers that can be several micrometers long and are required for twitching motility (Craig et al., 2004; Pelicic, 2008; Harding et al., 2013). Given this association between the type IV pili and twitching motility, it is not surprising that visualization of pili on necrotizing fasciitis strains revealed numerous long pili exceeding 2 µm on the surface of NFAb-1 and NFAb-2 cells (**Figure 4**). Given that the type IV pili system has been found to contribute to transformation as well (Harding et al., 2013), these elongated pili produced by NFAb-1 and NFAb-2 may also be a contributing factor to the accumulation of antibiotic resistance coding genes by these XDR pathogenic strains. Together these observations suggest that type IV pili may have the potential to contribute, at least in part, to virulence of bacterial pathogens, and may be particularly important for the less common presentation of *A. baumannii* necrotizing fasciitis infections.

An important component of the type IV pilus is the major pilin subunit, PilA, which, as its name suggests, is the most abundant pilin present in type IV pili (Harding et al., 2013). Apparent production of PilA was found in NFAb-1 and NFAb-2 (**Figure 4**), which matched expectations given the twitching motility phenotypes of these isolates. Since ATCC 17978 does not display twitching motility but produces PilA, twitching motility may be facilitated or regulated by additional factors that have not yet been described. Work in the well-studied *Pseudomonas aeruginosa* type IV pili system identified a regulatory system, Chp, that controls both twitching motility and cAMP intracellular levels (Whitchurch et al., 2004; Bertrand et al., 2010), as well as a number of proteins that control transcription of PilA and affect twitching motility (Buensuceso et al., 2017). These observations provide evidence that multiple layers of regulation may govern bacterial PilA production as well as twitching motility potentially explaining the lack of twitching motility by the ATCC 17978 strain, which produces PilA. These control mechanisms may be mediated by metabolic or transcriptional control processes, some of which could be represented in the NFAb genes identified during our comparative genomic analyses (**Figure 2** and **Supplementary Material**).

While biofilm formation may contribute to increased antibiotic resistance and could contribute to increased virulence, it did not appear that biofilm formation was responsible for necrotizing fasciitis pathogenesis as NFAb-1 or NFAb-2 produce only a moderate quantity of biofilm while their cellular toxicity is high. The CsuABABCDE type I pilus assembly system expressed by ATCC 19606^T^ cells is required for attachment to and biofilm formation on plastic surfaces (Tomaras et al., 2003; Tomaras et al., 2008). The PrpABCD type I pilus assembly system expressed by ATCC 17978 is required for surface motility and pellicle formation but negatively affects biofilm formation on plastic by bacteria cultured in darkness (Wood et al., 2018). The Wood et al. report (Wood et al., 2018) also indicates that ATCC 17978 may express adhesion and biofilm biogenesis functions that remain to be identified and characterized. Considering these observations and the widely accepted concept that biofilms play a role in bacterial virulence, it is possible to speculate that bacterial adherence and biofilm formation, even at the relatively low levels displayed by the NFAb strains, could play a critical role in their XDR properties and the pathogenesis of necrotizing fasciitis. Certainly, the role of these bacterial functions in the virulence of the NFAb strains should be confirmed once proper isogenic derivatives are available.

Bacterial virulence can also be modulated by a pathogen’s ability to enter host cells, wherein the pathogen may be able to evade host immune responses or antimicrobial drugs. As it pertains to *A. baumannii*, initial interactions with and adhesion to human epithelial cells are mediated in part by OmpA (Choi et al., 2008; Gaddy et al., 2009). Following adhesion, bacteria can enter the host cell and persist, where they eventually induce host cell death and potentially further dissemination into additional tissues or sites of infection (Smani et al., 2012; Smani et al., 2013). Both necrotizing fasciitis strains demonstrated their ability to invade human epithelial cells, with significantly more NFAb-2 bacteria entering the host cells during the 2-hour infection period than that observed for NFAb-1 bacteria (**Figure 6**). At first glance, this seems at odds with the A549 human alveolar epithelial cell viability data (**Figure 5**) where NFAb-2 was found to have a weaker effect on A549 cell viability than NFAb-1. However, no evidence for intracellular replication has been observed, so it is possible that entry into host cells stops or slows bacterial replication, resulting in less overall intracellular bacterial counts. The inability of ATCC 19606^T^ to invade A549 cells under the experimental conditions used in this study may also seem at odds with A549 cell viability data; however, it should be noted that other virulence factors such as two iron-regulated phospholipase C proteins have been demonstrated to impact A549 cell viability through cytolysis (Fiester et al., 2016).

There are few known virulence-associated genes unique to NFAb isolates as compared to the ATCC strains; some of which could contribute to the virulence of the NFAb isolates. One of these genes codes for a predicted crossover junction endodeoxyribonuclease RusA protein, which is involved in DNA repair and homologous recombination, functions that may be involved in the genetic plasticity of NFAb isolates. Also unique to NFAb strains are the modification methylases *Bsp*RI (M *Bsp*RI), *Dpn*IIA (*Dpn*M) and *Rsr*I (M *Rsr*I) as well as the mRNA interferase RelE and the structurally similar YafQ protein, which could be responsible for unique expression profiles in these isolates (Pedersen et al., 2003). These profiles could be due to transcriptional and post-transcriptional regulation that have been linked to controlling the functional expression of virulence factor genes (Bertram and Schuster, 2014) such as those involved in the promotion of bladder colonization (Norton and Mulvey, 2012) and switching between motile and non-motile biofilm forming phenotypes of uropathogenic bacteria (Hadjifrangiskou et al., 2011). There is increasing evidence showing the role DNA methylation plays in the regulation of gene expression (Beaulaurier et al., 2019), including the regulation of the expression of virulence factors. Some studies showed competition between MTases and transcription factors thereby affecting gene transcription (Van der Woude et al., 1996; Wallecha et al., 2002; Lim and Van Oudenaarden, 2007; Low and Casadesus, 2008) and thus the regulation of important clinical phenotypes such as biofilm formation, immune evasion, antibiotic susceptibility and virulence (Atack et al., 2015; Brockman et al., 2017). A similar function could explain the XDR and virulence phenotypes of the NFAb isolates described in this report. It is therefore a possibility that the phenotypes exhibited by NFAb isolates, especially those associated with type IV pili such as twitching motility, are a result of epigenetic modification in the form of DNA methylation.

One additionally interesting consideration regarding the clinical NFAb isolates described in this report is the temporal and spatial relationships between them. While NFAb-2 was collected postmortem from tissue which represented the initial site of infection, NFAb-1 was isolated from the blood during the course of the infection. In general, NFAb-1 demonstrates a more virulent phenotype, with increased motility, impact on host cell viability, and lethality in the *G. mellonella* model when compared to the NFAb-2 strain. This begs the question if the infectious strains of *A. baumannii* became less virulent over the course of the infection, from NFAb-1 to NFAb-2, perhaps as a response to the decreased need for the expression of virulence factors as the patient’s immune response deteriorated, or if the NFAb-1 strain actually acquired greater virulence which allowed for escape from the original site of infection where NFAb-2 remained. However, it is also possible that the more virulent phenotype of NFAb-1 was acquired following growth in the bloodstream as other studies have found that blood isolation or growth in human serum correlates with increased motility and PilA expression, respectively (Jacobs et al., 2012; Vijayakumar et al., 2016). Taken together, these observations demonstrate the genetic plasticity of these isolates potentially due to the presence and expression of genes coding for the aforementioned crossover junction endodeoxyribonuclease RusA, methyltransferases and mRNA interferases. While it could be argued that the NFAb-1 and NFAb-2 strains individually infected the same patient leading to necrotizing fasciitis, and therefore NFAb-2 did not descend from NFAb-1, this seems unlikely considering the rarity of this type of etiology, the similarity between the antibiotic resistances between these two isolates and the fact that the genomes of NFAb-1 and NFAb-2 are 98.28% similar. Future in depth study into the relationship between these isolates may provide crucial insight into *A. baumannii* gene regulation during the course of a necrotizing fasciitis infection and potentially many other infectious presentations.

To date, we present the first comparative genotypic and phenotypic analyses between commonly used *A. baumannii* ATCC clinical strains and isolates obtained from a fatal case of necrotizing fasciitis. Our data demonstrate significant twitching motility, biofilm formation, impact on host cell viability, intracellular survival and overall virulence of the NFAb-1 and NFAb-2 isolates. The virulence phenotype differences with the ATCC strains correlate with the finding that nearly 1,000 genes were present only in the necrotizing fasciitis strains, many of which could code for the distinct pathogenicity of the NFAb isolates and the clinical manifestations of the fulminant infection they caused in a compromised patient.

## Data Availability Statement

The original contributions presented in the study are included in the article/**Supplementary Material**. Further inquiries can be directed to the corresponding author.

## Author Contributions

JG, BA, MR, RC, SA, AT, RR, LA, and SF contributed to the conceptualization and experimental design of the study. JG, BA, MR, RC, JB, EO MM, CW, SA, AT, RR, and SF performed experiments. JG, BA, MR, RC, JB, EO MM, CW, SA, AT, RR, LA, and SF validated experimental design and provided experimental analysis with AT, SF, and BA specifically performing statistical analyses. JG, BA, MR, RC, AT, RR, LA, and SF contributed to drafting the manuscript. JG, LA, and SF provided resources for experiments, and JG, BA, LA, and SF provided supervision for the overall project. JG and BA contributed equally to the enclosed work. All authors approved the submitted version after participating in reading and revision of the draft compiled by JA, BA, LA, and SF. All authors contributed to the article and approved the submitted version.

## Conflict of Interest

The authors declare that the research was conducted in the absence of any commercial or financial relationships that could be construed as a potential conflict of interest.

## Supplementary Material

Supplementary Figure 1 Macrocolony biofilm assays. LB agar plates supplemented with Congo red, Coomassie brilliant blue and collagen I were inoculated with 5 µl of culture from each *A. baumannii* strain. Plates were incubated statically for 24 h at 37°C. The results were observed and recorded using a USB 2.0 Digital Microscope.
