# Supplementary figures and images for: Two Acinetobacter baumannii Isolates Obtained From a Fatal Necrotizing Fasciitis Infection Display Distinct Genomic and Phenotypic Characteristics in Comparison to Type Strains
Source: Front Cell Infect Microbiol. 2021 Apr 12;11:635673. doi: 10.3389/fcimb.2021.635673 (PMC8072282; doi:10.3389/fcimb.2021.635673)

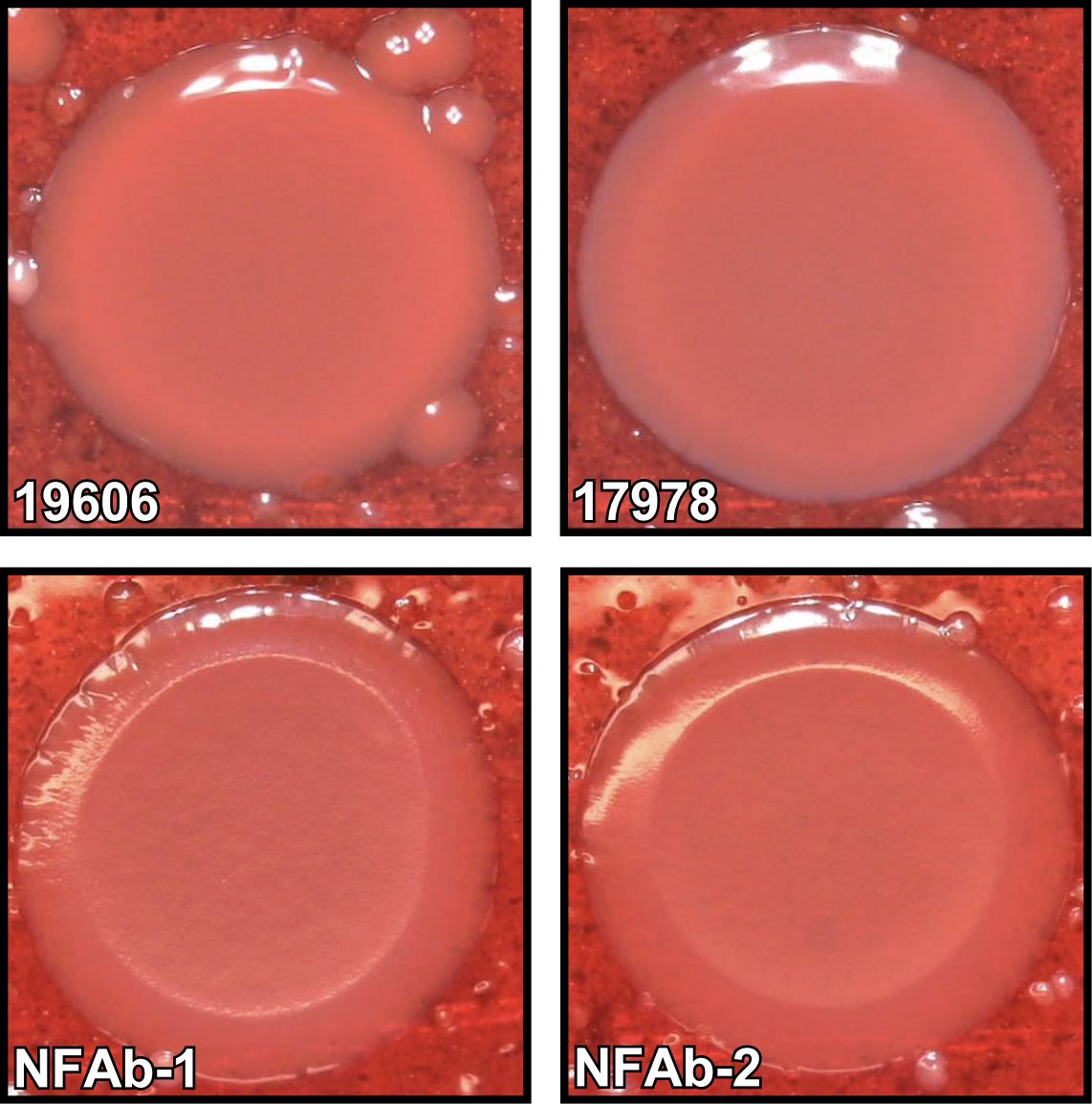

Supplement: Supplementary Figure 1 — Macrocolony biofilm assays. LB agar plates supplemented with Congo red, Coomassie brilliant blue and collagen I were inoculated with 5 µl of culture from each A. baumannii strain. Plates were incubated statically for 24 h at 37°C. The results were observed and recorded using a USB 2.0 Digital Microscope. [file Image_1.tif]
